# What Is to Be Expected from Heterogeneous Catalysis in the Pipeline to Circular Economy?

**DOI:** 10.1002/cssc.202402064

**Published:** 2024-11-27

**Authors:** Michele Melchionna, Paolo Fornasiero

**Affiliations:** ^1^ Department of Chemical and Pharmaceutical INSTM UdR University of Trieste Via Licio Giorgieri 1 34127 Trieste Italy; ^2^ ICCOM-CNR URT Trieste Via Licio Giorgieri 1 34127 Trieste Italy

**Keywords:** heterogeneous catalysis, sustainability, single atom catalysts, inorganic

## Abstract

Modern society requires a change in the philosophy of doing science, which faces the enormous challenge of being compatible with the new sustainability principles. Inorganic chemistry holds the keys to accelerate the transition given that most chemical processes or technology devices rely on the use or integration of inorganic materials. In particular, heterogeneous catalysis has a central role in promoting the transition from a linear economy to a circular one. To accomplish this, it is imperative that the modern schemes for catalysis will adopt a holistic approach based on sensible choice of raw materials, reliance on clean energy inputs and establishment of a robust framework of resource use and recovery. Some of these concepts are analysed here and discussed in Ref. [to a few selected examples.

## Graphical abstract

Heterogeneous catalysis based on the use of inorganic materials is required to adapt to our fast‐changing planet, and embrace fully the concept of sustainability and circular economy. The new drive takes on many interconnected aspects, from use of raw materials to assembly of smart catalysts.

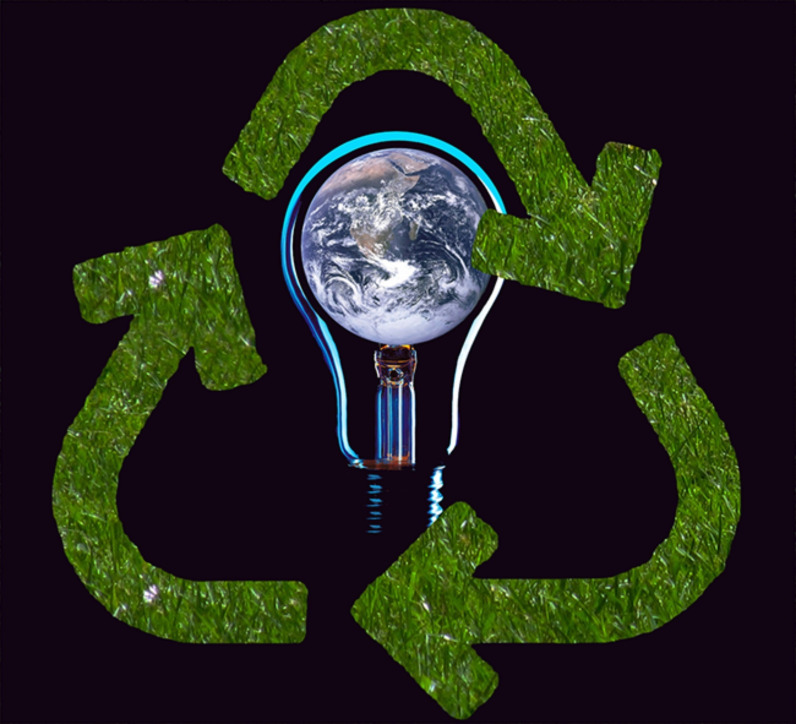

## Introduction

### The Circular Economy Context

For a very long time the worldwide production of commodities has been based on a linear economy, adhering to the philosophy of “use and disposal”, which had its peak in the first half of the 20^th^ century. In recent years, there has been a strong reaction, in particular by the United Nations, to the established hyper‐consumerism paradigm, promoting a new attitude by producers and consumers to embrace sustainability principles. This has emerged as an immediate and imperative necessity, in response to the worryingly increasing levels of pollution in all types of environments (air, water, soil) accompanied by a very steep increase of the average temperature of Earth in the last two decades. Responsibility of such phenomena is to a large extent accounted on anthropological activity in the aftermath of the industrial revolution, causing social inequality, geopolitical crisis, poverty and inacceptable healthcare standards among many populations in the world. The current vision is to abandon the linear economy and move to what is today called *circular economy*. This new framework has taken concrete shape through the multiple governmental regulations, summarized in the United Nations Sustainable Development goals. From a product production and manufacturing perspective, it is based on sustainability as the beating heart to develop new types of industrial processes. However, circular economy is a highly complex mechanism, with a wide spectrum of inter‐correlated types of action being mandatory. The transition of chemical processes from a high energy‐intensive regime to greener and less invasive approaches is one of the pillars of the new circular economy structure. Heterogeneous catalysis represents one of the areas that can give a large contribution to the implementation of sustainable schemes for chemicals production and therefore assist the economy transition. It turns out that the lion share of all catalytic materials is of inorganic nature (e. g. based on metals), and at first sight the sustainable management of inorganic substances may involve more complex schemes as compared to the organic counterpart. For this reason, inorganic chemistry is expected to overstretch towards innovative concepts for catalytic applications, getting out of the traditional research comfort zone and complete a chemical revolution started in the last decade and that yet to reach full maturity. Here we provide a critical outline of some of the most essential areas where inorganic chemistry for catalysis is in our opinion demanded to act upon, in order to facilitate the problematic transition to circular economy.

### Inorganic Materials and Sustainability

The type of inorganic materials used by industry, their abundance and distribution on Earth is one aspect to be carefully considered in developing catalytic materials and processes. Each element of the periodic table has a specific abundance, and in most cases the evaluation of sustainability levels of a particular process aims at avoiding use of rare or strategic elements. We believe that such an attitude is too superficial and does not provide the best guidance to address materials sustainability, as explained below.

A recurrent objective in homogeneous and heterogeneous catalysis is the search for catalytic metals other than the so called *noble* (or otherwise referred to as *precious*) *metals*. The latter is a list (more or less defined) of a few metals whose high price and scarcity create considerable sustainability issues. It is unquestionable that their use in catalyst formulation requires substantial reduction or even complete replacement. However, precious metal catalysts often exhibit superior activity, making their replacement challenging. Platinum (Pt) is one of the star metals for promoting a variety of processes, being a ubiquitously explored metal. Electrochemical applications such as oxygen reduction reaction (ORR), fuel cells and water electrolysis enormously benefit from the excellent conductivity, resistance, stability and fast kinetics of Pt;^[1]^ on the other hand, photocatalysis, thermal catalysis, drug discovery, organic synthesis have been heavily relying on Pt catalysis for many years.^[5]^ Other noble metals such as palladium, gold, iridium, rhodium and silver have also shown prominent catalytic uses, as well as a versatility and stability which is hard to match with other transition metals. For example, palladium (Pd) has a dominant role in organic coupling reactions,^[12]^ while gold (Au) is the most investigated metal for activation of double and triple C‐C bonds,^[14]^ being also very attractive when in form of size‐precision nanoparticles because of its tuneable plasmonic properties.^[19]^ Apart from the intrinsic low abundance, a stronger connection of precious metals to the emerging concepts of sustainability originates from the uneven geopolitical distribution of the raw minerals from which they are extracted. This aspect has been the seed of a long and sad history of national or international conflictual episodes for the control of mining and export. Not only precious metals are concerned, but also abundant metals with specific geographical location. For some of these metals, the high technological value has been a plague for the producing country. Notorious examples include the strong social and ethical connotations in the Republic of Congo relative to the collection of *coltan* (the raw mineral for Tantalum and Niobium extraction, two important elements in the industry of semiconductors, computers and mobile phones) or cobalt (essential component of mobile phone batteries), or the irresponsible mining of nickel, lead and other metals in some regions of Siberia that has caused great health and environmental crisis to the population and territory.

Another class of relevant inorganic materials that has problematic production schemes is that of *rare earth elements* (REE). These include the lanthanides, yttrium and scandium, and find application in various processes which demand a high levels of purity. However, given the inherent occurrence of REE as mixtures of various elements with similar physical properties, the separation procedures are technically very challenging. Moreover, geopolitical factors are as usual affecting the level of criticality of REE, as demonstrated by the REE export restrictions that were established by the Chinese government between 2007 and 2010.

Reflecting on the above facts and examples, material management must go well beyond the precious metal avoidance. The modern material sustainability has taken a pictorial shape around maps of element criticality, in which REE, precious metals and in general all materials that have applicative fields have a certain level among the list of “critical raw materials” (CRM). As mentioned above, defining a level of criticality for each element is a very complex matter, because it must take into account a number of factors, some of them dependant on time. The main classification made by the EU uses two criteria: 1) economic importance and 2) risk of supply disruption

**Figure 1:**
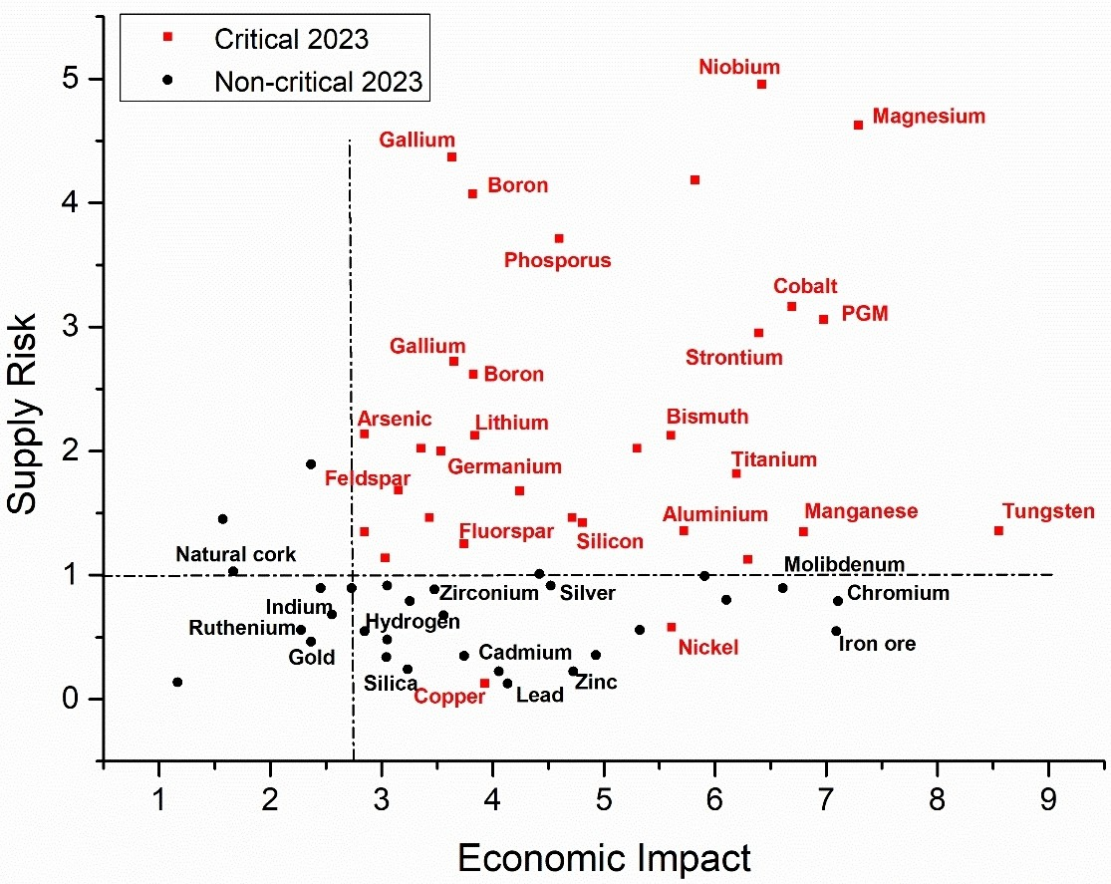
Critical raw material map as outlined by the European Commission, adapted with permissions from “European Commission, Study on the Critical Raw Materials for the EU 2023 – Final Report”. Dotted lines indicate EU thresholds in Economic Impact and Supply risk.

Figure 1 is a graph taken from the EU 2023 Final Report on Critical Raw Materials, and a few unexpected results can be observed. Surprisingly, gold is not indicated as critical based on the defined criteria, while other “abundant elements” such as phosphorus, magnesium and bismuth are identified as CRM. We note that a list of critical elements (or in general materials) based only on economic considerations is obviously unsatisfactory, and a more meaningful roadmap to circular economy requires the evaluation of a number of additional ethical points. Two of these concern the toxicity of the catalytic material, and the possibility and methods for recycling the spent catalysts. Nevertheless, the graph is a useful testimony about the complexity of modern material management, and leads to our underlining fundamental question: is chemistry truly developing towards the best circular economy? From a glance to a big body of recent literature, it appears that academic research still struggles to adapt to a fast‐changing world, dominated by fluctuating politics and population needs. We wish to illustrate as a representative example the great deal of attention drawn by the replacement of precious plasmonic metals such as gold with more available plasmonic elements such as bismuth and copper.^[22]^ Both Bi and Cu are intriguing metals, and their plasmonic characteristic is surely worthy being investigated, but from a glance at the CRM map, this research should be justified on account of scientific interest, rather than on economic terms. From an economic point of view, new types of plasmonic materials such as TiN have emerged as better alternatives.^[25]^

Our conclusions regarding this section is that a new framework for materials sustainability is to be established, underlying a complex mechanism based on manifold principles and sub‐schemes. In fact, the hurdles are so many to cause initial discouragement. However, inorganic materials will have to abandon the comforts of traditional and well‐established schemes, become an adapting machine that explore new territories guided by a set of principles which are outlined below in general terms.

### Dematerialization of Catalysis.

The concept of *dematerialization* involves the lowering of the total amounts of used materials for a catalytic process (or any technology process in general) adhering to the principle of atom economy. Substitution of CRM with more available elements is a first step, but insufficient by itself. An interesting strategy is to improve the functionality of the material in relation to the specific catalytic reaction. Tailoring the functionality of the catalyst often determines an enhancement of the intrinsic activity, and expectedly most of the research in catalysis deals with catalyst design. The reduction of the size (and morphology) of catalytic materials to the nanoscale is the supreme example of increasing functionality. The underlying immediate advantage is that moving from bulk to nanoparticles, the surface area becomes larger, implying a bigger number of active sites with equal mass of the catalyst. This aspect appears of great importance for the concept of *dematerialization*, meaning the employment of less material to drive chemical processes with comparable efficiency, one of the main principles of circular economy frameworks.^[29]^ Still, nanostructuring introduces significant differences in the material properties, and sometime this reflects into unexpected results in terms of activity, selectivity and stability. In general:

1. considering that the surface area to volume ratio (S/V ratio) considerably increases, the coordinative surrounding of surface atoms become progressively more critical. Therefore, a substantial variation of catalytic selectivity may result from the comparable proportion of surface sites with different coordination environments, originating from diverse types of interaction with adsorbates.
2. If size reduction proceeds to the level of a few nanometers, the electronic properties of the metal can be profoundly altered, due for example to quantum confinements, leading to a change of catalytic performance.^[30]^
3. Higher S/V ratios also imply higher surface energy, favoring aggregation of the nanoparticle under operative conditions. As a result, the performance may vary over time, thus affecting the stability of the catalyst.

An additional point concerns the scalability of the process in the production of nanomaterials. The synthesis of nanomaterials with sufficient homogeneity (particle size, morphology, composition homogeneity, distribution of defects etc.) often requires specific conditions and additives, coming to a cost which is not always simple to sustain at industrial level.

**Figure 2:**
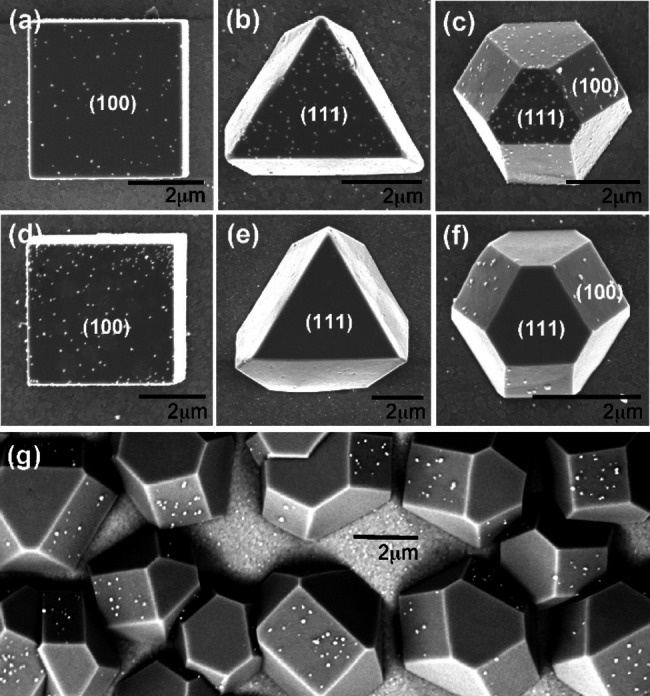
SEM images of Au particles deposited on Cu_2_O crystals without (a–c) and with sodium dodecyl sulfate (SDS) (d–g). Reprinted with permissions from Ref. [14]. Copyright (2009) American Chemical Society.

It has to be concluded that the incessant (and indeed justified) search for nanostructuring of metal catalysts cannot be the most general solution. In fact, accurate analysis with regard to the specific application, and all the key mechanistic features and structural evolution of the catalyst could reveal counterintuitive results. In any case, the use of nanotechnology for sustainability remains topical, in particular for catalysis, where the concomitant minimization of CRM loadings and improvement of performance represents a powerful tool for approaching circularity of chemical processes. In some notable examples, use of precious metal catalysts such as Pt could be reduced to an ultra‐low content by reducing the size of Pt to nanodots of ca 2 nm, with a boosting activity in water electrolysis. But to achieve appealing performance, the formulation of the electrocatalyst often requires integration of additional components to suitably tailor the properties of the catalytic ensemble.^[33]^ Over the years, the rationalization of methodologies to control size, shape and specific site‐deposition of nanoparticle catalysts has been evolving, addressing the particular requirements of the different catalytic reactions.^[34]^ As a representative example, Rej *et al*. comprehensively reviewed the synthetic strategies to control the size, faceting and structural evolution of nanostructured Cu_2_O, an appealing photo‐ and electro‐catalyst for several important transformations such as CO_2_ reduction and H_2_ evolution.^[36]^ Such a control can have a direct impact on the improvement of catalytic performance, as the different facets have specific adsorption energies towards different substrates; on the other hand, the catalytic activity can be affected indirectly, as the shape and faceting regulate the deposition of other active metal NPs on selective planes. Figure 2A shows examples of morphology control on Cu_2_O nanoparticles that results in different activity towards azide–alkyne cycloaddition,^[37]^ while in Figure 2B, SEM images demonstrate the specific deposition of Au nanoparticles on particular facets of various Cu_2_O NP morphologies.^[38]^

One modern avenue to maximize the dematerialization of catalytic processes without sacrificing activity is the employment of the so‐called single atom catalysts (SACs). SACs consist of isolated metal atoms dispersed on suitable heterogeneous supports, which have been attracting great attention in view of their unique catalytic potential, because in principle they could lead to a 100 % metal atom utilization. The absence of any adjacent metal atoms has profound effects on the mechanism for several reactions because of the restricted binding mode of the reactant, so that selective routes are more favored. SACs have been fruitfully used in the electrochemical activation of small molecules such as CO_2_, O_2_ and N_2_, where the single atom nature of the catalytic metal is instrumental for the selective binding mode of the reacting molecule (and consequently reaction routes).^[39]^ The most meaningful case study is the selective electroreduction of O_2_ to H_2_O_2_, where the binding of the O_2_ molecule onto the metal is restricted to an end‐on configuration. Hence, the rupture of the O‐O bond arising from the side‐on binding on two adjacent metal atoms is avoided.^[40]^ cost effectiveness of SACs is particularly attractive when the catalytic system is based on precious metals, which indeed have a dominant role in SAC catalysis.^[41]^ However, the synthesis of SACs is highly challenging, as typically too small metal loadings are viable, as larger contents easily cause aggregation into small clusters or nanoparticles, and the single atom nature is partly lost. The resulting mixed SAC/nanoparticles generate large uncertainty in the understanding of the mechanism. Nevertheless, ingenious synthetic strategies are continuously devised to increase as much as possible the single atom loading. A representative work was reported by Li *et al*. who could achieve high concentration of single Pt atoms, as high as 24.8 at %, supported on CuS_x_ nanospheres by means of a ion exchange method. The catalyst could be incorporated into a an electrochemical device producing H_2_O_2_ with the impressive rate of 546±30 mol kg_cat_^−1^ h^−1^.^[44]^

**Figure 3:**
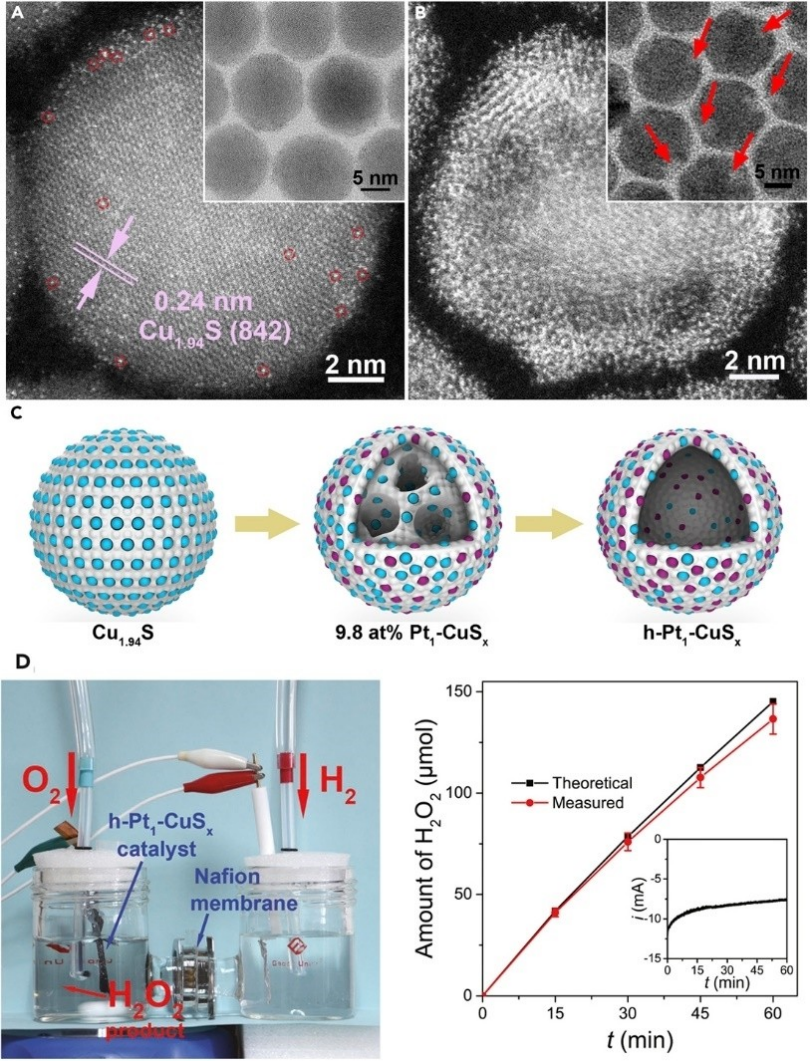
A–B) TEM images of Pt_1_‐CuS_x_. C) graphical sketch of the synthesis. D) The electrochemical device used for the electrochemical synthesis of H_2_O_2_ and chronoamperometric profile. Adapted from Ref. [18]. Copyright (2019), with permission from Elsevier.

H_2_O_2_ electrosynthesis becomes even more attractive when the precious metals are replaced by abundant metals, and available examples in literature with cobalt, nickel, copper and iron are numerous (Figure 3). Typically, Co is top ranked for the 2‐electron ORR process on account of the optimum d‐band position.^[45]^ On the other hand, Cu SACs raise great expectations in organic synthesis, where they are used to accomplish valuable types of conversion, such as click chemistry reactions,^[47]^ oxidative couplings,^[48]^ or other photocatalytic conversion.3, 4

**Figure 4:**
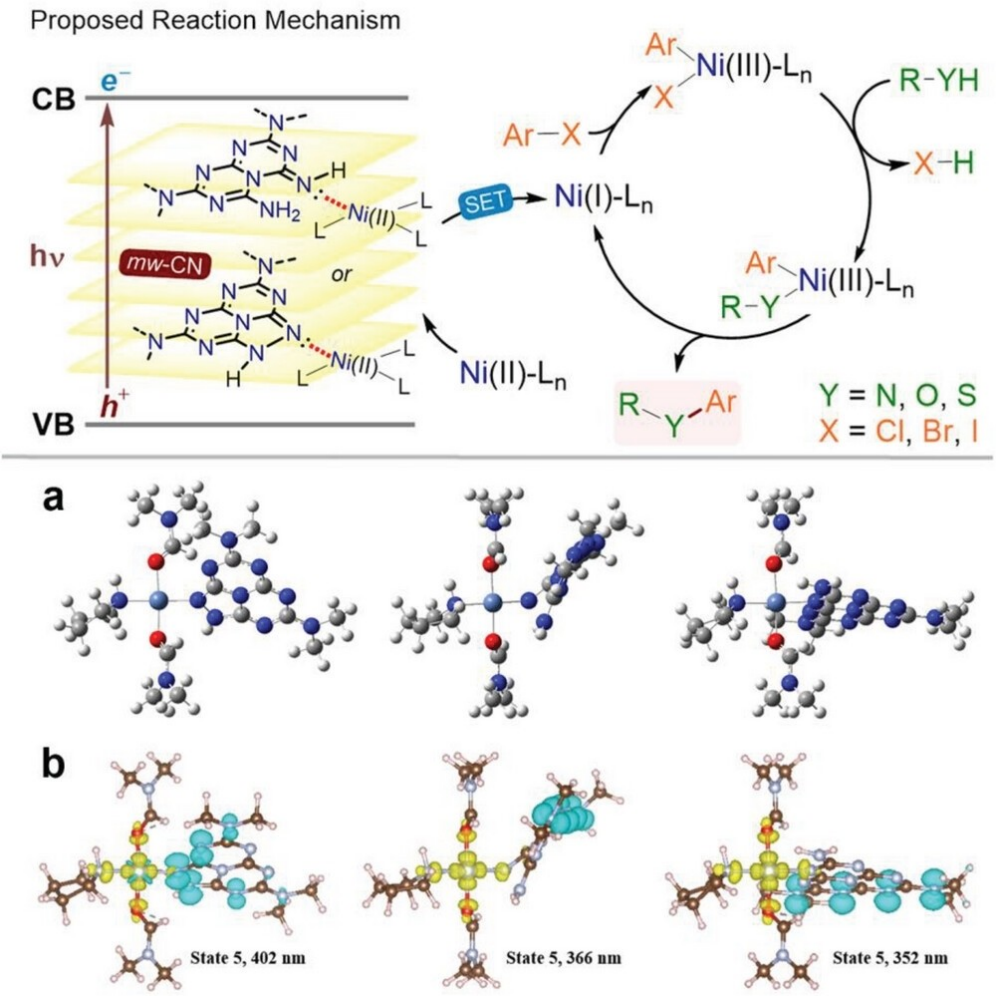
Dual photoredox catalysis mechanism and (time‐dependent) DFT calculations. Top panel: sketch of the proposed mechanism. a) DFT‐optimized geometries of Ni complexes. b) Attachment/detachment densities for selected excited states in the Ni complexes. Reprinted with permissions from Ref. [22] under the terms of the Creative Commons CC BY license.

An innovative opportunity that could address some of the above issues with conventional SACs is to exploit the SAC properties only transiently, devising a system where a “bind and release” mechanism can be implemented within the catalytic route (Figure 4). For instance, our group engineered the structure of graphitic carbon nitride (g‐CN), a very popular metal‐free semiconductor, with appropriate functional groups which could bind a nickel center with optimum binding energy. During the course of the photocatalytic C‐X coupling reaction (X=N, O, S), the electron transfers between g‐CN and the bound Ni made the oxidation state of the metal oscillating, and when in oxidation state (I), the binding energy was disfavoured and the Ni(I) was released from the g‐CN surface, carrying out the catalytic cycle in solution.^[49]^ During the redox cycle, however, the return to the oxidation state (II) could guarantee continuous re‐binding to the g‐CN, allowing the key step of the electron transfer. This strategy is actually likely to occur in many of the so‐called *dual photoredox catalysis*.^[50]^ However, the bind and release feature is seldom recognized and thoroughly investigated, even though mastering its dynamics could lead to the catalyst design where some of the problems with well anchored SAC are mitigated. This may be considered one example of smart materials, which will be further discussed in section 3.

As an ultimate dematerialization step, moving into complete replacement of any metal component with non‐metal abundant elements such as carbon, nitrogen, oxygen, sulphur, phosphorus. Once again, catalysis is the elected demonstrator of this principle, and the spectrum of reactions triggered by non‐metal catalysts is growing wider and wider. The above discussed g‐CN is also able to trigger various types of organic conversions without the aid of any metal, provided that the structure of the catalyst is opportunely tuned to fulfil the specific reaction requirements.^[53]^ The 2‐electron ORR can also proceed readily with opportunely doped carbon structures,^[57]^ sometimes even surpassing activity of metal catalysis.^[58]^

### Functional Multi‐Phase Materials

**Figure 5:**
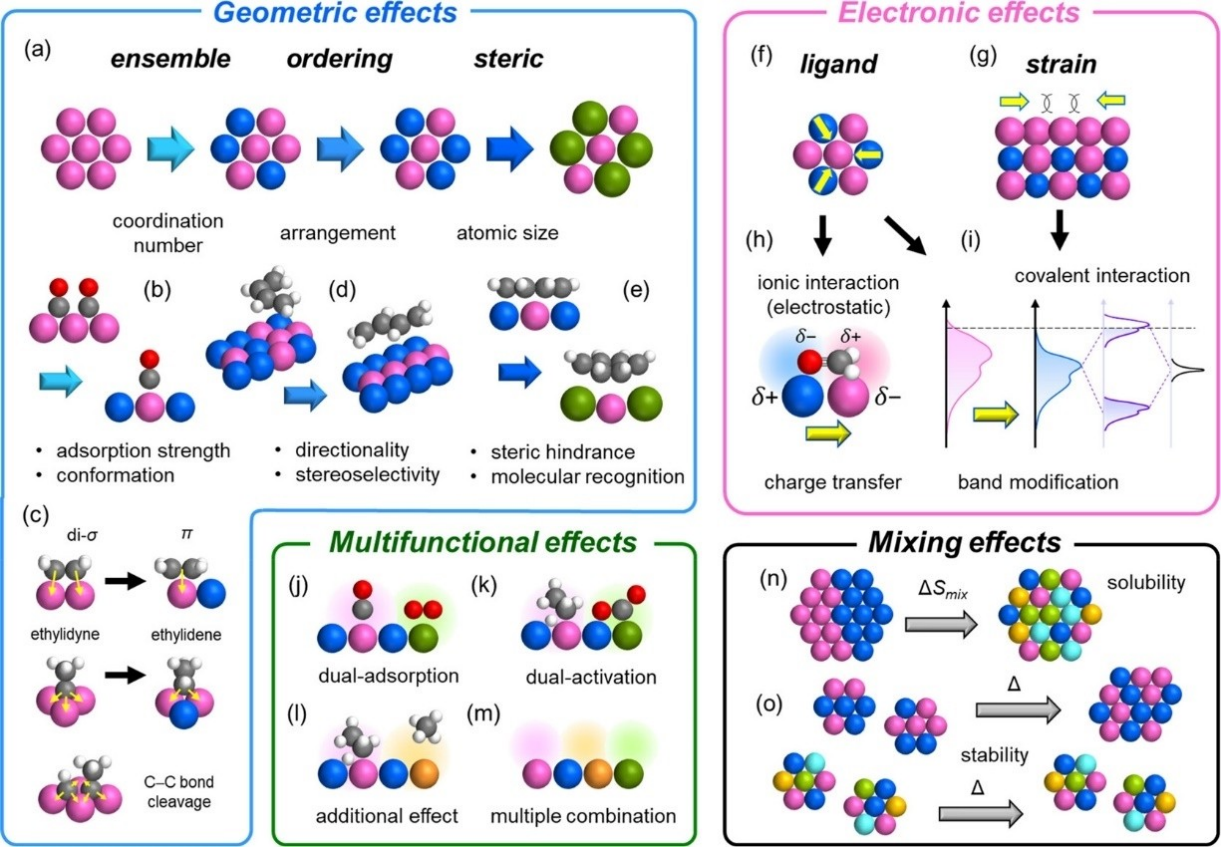
Summary of alloying effects on catalysis. Reprinted with permissions from Ref. [27]. Copyright (2022) American Chemical Society.

Where the dematerialization of chemical processes is very difficult to achieve, an insightful look should focus on ways to make the material so efficient for the desired application, that any cost is overcompensated by the achieved performance. The economic value is then accompanied by less wasteful use of resources, and the process can be a level up in the sustainability scale. We present here some of the general strategies used in heterogeneous catalysis to improve performance to a high level of efficiency. The most powerful strategy to increase the efficiency is to combine two or more components and assemble multi‐phase materials where the interplay between the different phases (which may occur concomitantly or sequentially) gives rise to synergistic effects. Most famously, metal alloys are well‐known types of materials ubiquitously employed in various applications, including catalysis.^[62]^ They offer the enormous possibility of changing the electronic states and/or the geometric environment of the active metal center. It should be pointed out that the origins of the “alloying effects” are complex to tackle and not always fully understood,^[63]^ often requiring many types of advanced characterization studies. Several possibilities arise and at times there is a combination of the different effects, which are difficult to evaluate individually (Figure 5).

Nevertheless, the utility of alloys is very practical as it results in alteration of the catalytic properties of the metal site. Importantly for sustainability, the efforts in preparing the alloyed catalyst are at times overcompensated by the considerable improvement in performance. For instance, in the hydrogen‐mediated *cis‐trans* isomerization of alkenes, the ordering effect of intermetallic RhSb (in contrast with pure Rh) proved to be instrumental to inhibit the undesired overhydrogenation reaction, thus attaining high selectivity.^[64]^ Charge transfer between the two metals can be predicted on the basis of electronegativity and work functions,^[65]^ although not always this phenomenon is simple to understand. Still, advantages from charge transfer in alloy catalysts have been well documented, exploited for example in chemoselective hydrogenation,^[66]^ hydrodeoxygenation^[67]^ or electrocatalytic oxidation^[68]^ reactions. Increasing the number of metal elements that constitute the alloy offers a higher level of multi‐functionality. As an emerging material that is attracting great attention, high entropy alloys (HEA) incorporate five or more elements mixed in similar proportions. In general, the thermodynamic of alloy formation governs the resulting final materials where the elements are either distributed with precise order (intermetallic compounds) or in a random fashion (mixed phases, or solid solutions). At the thermodynamic equilibrium, the structure with the more negative Gibbs free energy (we will call Δ_f_G the free Gibbs energy for formation of intermetallic compounds and Δ_mix_G the free Gibbs energy of solid solutions) will prevail. Because Δ_f_G=Δ_f_H−TΔ_f_S and Δ_mix_G=Δ_mix_H−TΔ_mix_S and given that Δ_mix_S is always larger than Δ_f_S, the final structure will depend on the relative values of Δ_f_H and Δ_mix_H (with respect to the terms TΔ_f_S and TΔ_mix_S. In reality, the situation for the formation of pure intermetallic compounds or ideal solid solutions is much more complex, and many factors intervene beyond entropic factors, leading to complicated phase arrangements. But in general, HEA formation is driven by a very large positive value of the configurational entropy, as it occurs in structures with many types (>5) of metal elements, which overcomes the enthalpic contribution.^[69]^ Despite a simplified (and not rigorous) justification for their formation, HEA have become a very popular class of material, where the high multi‐functionality arising from a large number of multi‐metal interphases, producing several notable effects. In particular, HEA′s strength retention, thermal stability, toughness towards fracture, resistance to corrosion and oxidation and other interesting mechanical properties have projected HEA as the new forefront materials in high‐temperature applications.^[70]^ HEA are also very appealing from the perspective of energy and environment. In environmental protection applications, they can be used as catalysts for degrading water pollutants, or as radiation protection materials, while in energy they can be used as gas storage (for example H2), capacitors and other energy storage devices and thermoelectrics. In catalysis, HEA can drive several processes, in particular for thermocatalytic^[71]^ or electrocatalytic reactions.^[73]^

**Figure 6:**
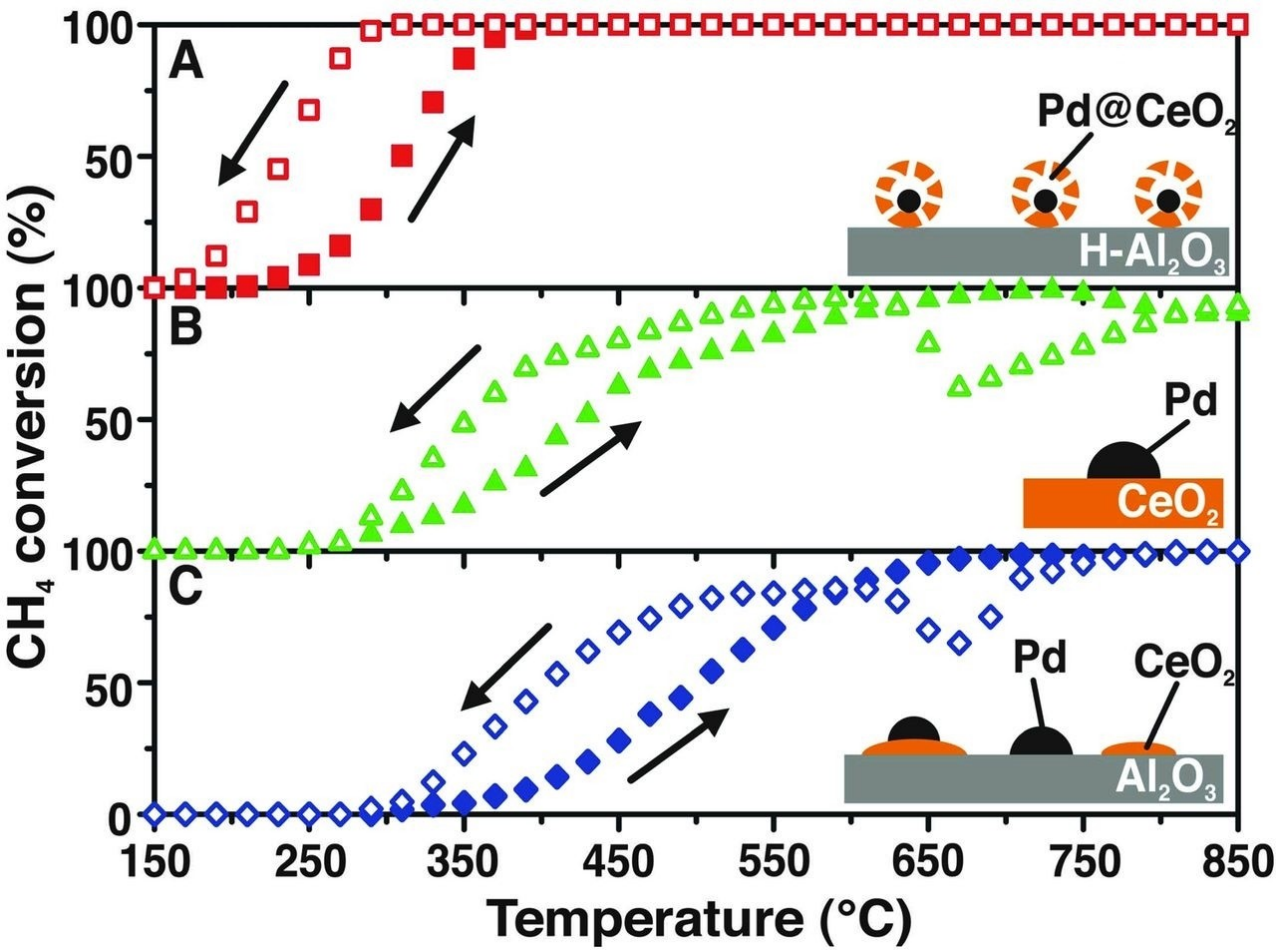
Heating and cooling during CH_4_ conversion for: A) Pd@CeO_2_/hydrophobic Al_2_O_3_ (H‐Al_2_O_3_) core–shell catalyst; B) Pd/CeO_2_ prepared by incipient wetness impregnation, and C) Pd/CeO_2_/Al_2_O_3_ prepared by coimpregnation of Pd and Ce precursors. From Ref. [40]. Reprinted with permission from AAAS, copyright 2012.

Hierarchical catalysts provide a great example where this concept is fruitfully exploited to drive difficult conversions with high efficiency. Here, the multi‐phase synthesis follows a well‐controlled order of assembly and the interface is built across precise surfaces. A seminal work by Yamada et al. described the bi‐dimensional successive layering of Pt and CeO_2_ nanocubes on flat SiO_2_ sheet. The interfacial double Pt–metal oxide formed allowed tandem catalysis for the sequential two‐step sequence of CH_3_OH decomposition to CO and H_2_ (Pt–CeO_2_), and the hydroformilation of ethylene (Pt–SiO_2_).^[77]^ The multi‐functionality of hierarchical materials represent a modern approach useful in many disciplines and for many applications. Inorganic chemistry is enjoying fast progresses in their development taking inspiration from biological systems, producing hierarchical hybrids and composites in a variety of morphologies for replicating specific mechanisms of action.^[78]^ In catalysis, higher activity, stability or selectivity can be achieved if the multifunctional material is wisely designed and synthesized, meaning that the different functions are integrated within the material to produce a system‐level upgrade of the characteristics with respect to the individual components. Our group reported the self‐assembly of precious metal nanoparticles and metal oxide in a core‐shell mode, and this type of structure proved to be ideal for considerably extend the lifetime of the catalyst during high temperature methane oxidation, as the porous oxide shell could protect the precious metal core from sintering. Moreover, the hierarchical architecture improved catalytic activity by exploiting synergy between the Pd and CeO_2_ phases (Figure 6).^[82]^ The strategy showed excellent versatility, and was successfully used in solid oxide fuel cell applications,^[83]^ water‐gas shift reaction^[84]^ and photo‐ and electrocatalytic H_2_ evolution and CO_2_ reduction,^[85]^ such processes being important in sustainability schemes.

The use of a protection shell was originally applied to the protection of metal nanoparticles from coalescence, and to control the metal electronic and optical properties. In this regard, layering approaches could generate highly homogeneous core‐shell structures made of Ag@TiO_2_ with tuned plasmonic properties^[87]^ while in Au@SiO_2_ the optical properties of gold could be controlled by varying the shell thickness.^[88]^ The core‐shell configuration is also applicable to metal/metal systems, for instance to change Raman scattering properties,^[89]^ or to metal oxide/metal oxide nanocomposites.^[90]^ In general, hierarchy is one principle adopted to steer the performance of inorganic materials. What is intrinsically most relevant from the applicative point of view is the fact that the materials’ chemical and physical properties can be largely altered at the interface between two components.^[91]^ For example, formation of Type II heterojunctions and Z‐schemes have become the modern solution to drive challenging photocatalytic reactions, proving particularly useful for the energy‐relevant H_2_ generation and CO_2_ conversion.^[92]^ This strategy capitalizes on the possibility of separating the two types of charge carriers (electrons and holes) thanks to the interfacial equilibration of Fermi level and subsequent built‐in internal electric field that allows coupling of charge carriers. As a representative example, CdS/WO_3_ heterojunctions were evaluated in the photocatalytic reduction of CO_2_ to CH_4_, and the authors demonstrated that the mechanism based on formation of Z‐scheme was responsible for the much larger productivity of the composite with respect to the individual catalysts.^[93]^

### Materials Go Smart

As discussed above, chemical processes embracing the circular economy philosophy have stimulated the development of multi‐functional materials, where specific functions are built within the structure of the material. The objective is to improve the performance in the target application while decreasing as much as possible material usage. Typically, multi‐functionality is achieved by opportunely combining two or more components, as seen earlier for high entropy alloys, or by judicious chemical or physical modification of the structure. A frontier concept that could give a notable boost at the sustainability of catalytic processes is to abandon the conventional “steadiness” of multi‐functional materials, and incorporate elements of smartness that make the material dynamic. In practice, in the final multi‐phase structure, the properties and functions can change in response to an applied external stimulus. Applications go well beyond catalysis, for instance piezoelectric materials are able to transduce mechanical deformation into a precise electric signal (or *vice versa*). The effect of piezoelectricity, discovered by the brother Pierre and Jacques Curie in 1880, is based on polarization of the material following a tensile or compressive stress, resulting in the generation of a voltage (direct piezoelectricity). The inverse process (indirect piezoelectricity) occurs from application of a voltage which produces a mechanical deformation.

**Figure 7:**
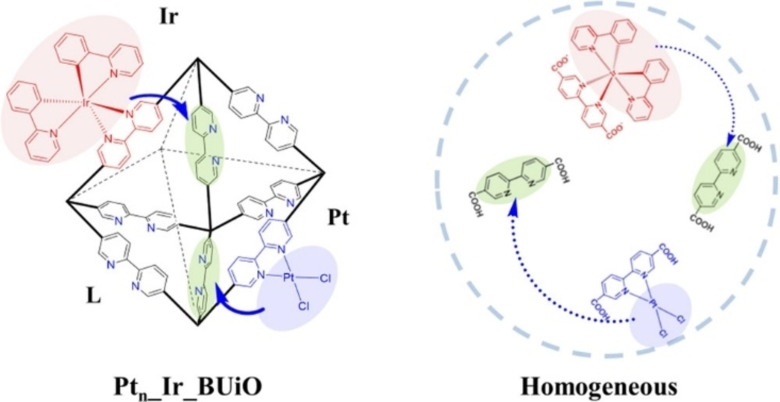
Graphical sketch of the operation Principle of Self‐Healing MOF (Pt_*n*__Ir_BUiO) and the Corresponding Homogeneous System. Reprinted with permissions from Ref. [53]. Copyright (2016) American Chemical Society.

In the context of smart catalysts, the principle of *self‐healing* represents a powerful strategy to mitigate deactivation problems, and it is based on the ability of the catalyst to self‐regenerate during operating conditions. Costentin and Nocera developed self‐healing Co phosphate electrocatalysts for water splitting taking advantage from the ability of the catalyst to self‐assemble in defined potential windows.^[94]^ Autonomous regeneration is also an innovative route to bypass the problem of photocorrosion of the catalyst during heterogeneous photocatalytic reactions. This problem arises typically with non‐noble metal catalysts, where leaching of the metal occurs, with releasing of inactive metal ions in solution. As a consequence, the drop of stability becomes a major obstacle in development of photocatalysts with intrinsic high activity. A self‐healing approach compensates for the leaching of the active metal phase by continuous redeposition of the metal under operative photocatalytic conditions; this approach, demonstrated to be viable with Cu catalysts,^[96]^ requires a careful evaluation of many parameters, above all a matching between the reduction potential of the specific leached metal and the band positions of the semiconductor support. The self‐repair mechanism in photocatalysis has also been adopted in homogeneous metal complexes taking inspiration from the PSII in plants. The artificial self‐healing platform consisted of a metal organic framework (MOF) bearing bipyridine groups within the linkers, able to continuously coordinate Pt(II) and Ir(III) centers during photocatalytic hydrogen evolution (Figure 7), achieving very long stability.^[98]^ The smartness of the catalysts addresses an issue of high relevance to the development of sustainable schemes of chemical production, because a too short lifetime of catalyst implies time and resource wasting, due to the need of continuous re‐synthesis of the catalyst.

The concept of switchability is another main tier of smart materials, whereby stimulus‐induced dynamic and reversible structural changes can offer a handle that can drive the selectivity of a chemical process. In the landscape of inorganic materials, perovskites represent a remarkable example of switchable materials, undergoing a process called ex‐solution. Here, during preparation of the perovskites (general chemical structure ABX_3_, where A and B are two metals with restricted requirements and X is a non‐metal such as oxygen or a halide) a foreign metal can be incorporated on the B side as a cation. Then, under reduction conditions, the metal cations can move inside out (and reversibly) of the perovskite lattice due to a change of the perovskite lattice, where the cell parameters vary with temperature. This feature was originally shown with Pd cations during fluctuations in exhaust‐gas of petrol engines enabled the dynamic structural responses and avoided the problem of Pd aggregation.^[99]^ Interestingly, the expansion of the lattice in some perovskites could be also triggered by extended time illumination, which led to improved solar cell applicability.^[100]^ Within our group we improved the *ex‐solution* process by adopting atomic layer deposition (ALD) of thin films of LaFeO_3_ on top of MgAl_2_O_4_ supported Pd NPs, which decreases the undesired aggregation of the nanoparticles during CH_4_ and CO oxidation reactions.^[101]^ Apart from stability, ex‐solution can be designed to switch the selectivity of catalytic processes, as we demonstrated for the ex‐solution of FeO_x_ nanoclusters from the inside of N‐doped carbon shell to the outside, with a significant switch of selectivity for ORR from a 2‐elctron to a 4‐electron reduction.^[102]^ Exploiting materials smartness in applicative fields can be a driving force towards a better managements of materials, resources an time. However, given the complexity of fabrication of smart materials, there is a possible risk that their development becomes itself untenable, due to the long stage of investigation, optimization and testing. Moreover, the correct use of a new smart materials must inevitably hinge on the full and in depth understanding of its properties, which requires a long and complicated characterization stage through a crosscheck of multiple and advanced techniques. In particular, if smart materials are to be accepted by industry and produced in large scale, their use in many cases may be unsustainable. Discussion on these aspects is expanded in the next section.

### Process Sustainability

A holistic circular economy related to inorganic chemistry should also carefully consider the downstream use of the assembled material. The problem of process sustainability is rather well‐known and it focuses on the change of the energy input to drive specific processes such as chemical reactions. The energy source to promote reactions was traditionally based on thermal energy, typically originating from fossil fuels, which is of very high environmental impact. Hence, new concepts are being adopted where the energy used is deriving from renewable sources, and therefore a decisive element of material formulation and synthesis is the compatibility with some process schemes employing green energy. The landscape of catalytic reactions is gradually changing according to these requirements, with a close up to reaction steps based on renewable energy inputs. It appears that the most conspicuous part of the research literature deals with the use of photocatalysis and electrocatalysis. These are useful types of catalysis can be applied in many fields, from chemical production to energy‐ and environment‐related reactions, to medical and biological applications, device assembly (e. g. photovoltaics) and so on.^[103]^ It would be impossible to give a short and comprehensive overview of the many types of materials for the many processes, therefore here we will highlight only some of the general potentials, criticalities and limitations. Light is a highly desirable energy source, particularly if its visible part of electromagnetic spectrum, which is the highest fraction irradiated by the sun, can be exploited. The design of catalytic materials, which generally are semiconductors, is conceived taking into account the optical characteristics of the material, specifically its energy band configuration.^[111]^ However, the complexity of the light‐induced phenomena and the interaction of the catalysts with the reactive surrounding very often prevent an economic convenience. Aspects such as fast recombination of electron‐hole pairs (the photo‐generated charge carriers), deactivation or photo‐corrosion of the catalytic material during operation, leaching of the active site, poor selectivity, poor interaction with the reagents, etc are notorious problems. The optical characteristics of the semiconductor such as bandgap energy and type (if direct or indirect) and position of the valence and conduction band imply a difficult balance: for instance, direct and narrow bandgaps absorb light more efficiently, but the rate of charge recombination is also very high. The type of application is then decisive for the most opportune choice. The most popular approaches to maximize the performance of a photo‐responsive compound is to carry out a detailed structure engineering. Typical avenues include: 1) introduction of dopants or defects to modulate band‐gap and band position with necessary precision, 2) use of metal co‐catalysts or multiphase semiconductor interfaces to extended charge carrier lifetime and adjust CB and VB energy levels, 3) control morphology and textural properties for better mass transport (diffusion of reactants at catalytic sites) and charge mobility, 4) introducing stimulus‐responsive functions or other smart elements for improved stability (self‐healing) and selectivity.[^53^, ^112^] The *ad hoc* design is then evaluated in the light‐driven process of interest; if the structure/activity relationship has been correctly studied, then the performance will guarantee a cost‐effectiveness which is attractive for industrial exploration. However, material design is only one step of the journey, as efficiency of the photocatalytic process depends on other parameters, too, which could be serious bottlenecks for realistic implementation. For instance, the photoreactor setup is a subject where dedicated studies must be devoted, so to allow for the best possible materials’ performance and avoid efficiency losses. The design requirements vary according to the specific applications, which define the particular geometry on the basis of the nature of reagents/products, the conditions, the mechanism, the kinetic.^[116]^ This may seem to require only engineer competences, but in reality the reactor also needs to adapt to the type of material, its structure and particle size, so that inorganic chemist′s know how is demanded.^[117]^

**Figure 8:**
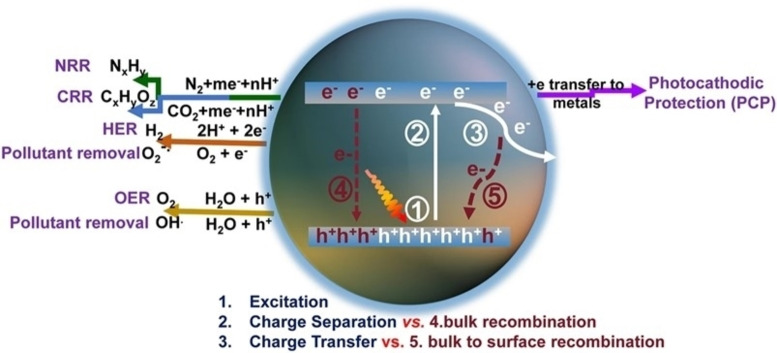
Schematic photoexcitation, charge transport, and solar applications of semiconductors. Reprinted with permissions from Ref. [63] under the terms of the Creative Commons CC BY license.

From the perspective of sustainability, cost‐effectiveness is not the unique goal. The type of process itself must be preferably in line with the general new philosophy of circular economy. For this reason, in the realm of catalysis, the most explored reactions are those with an impact in energy storage and supply or environmental remediation. Currently, the most investigated photocatalytic processes include (Figure 8):^[119]^

1. hydrogen generation,
2. production of carbon fuels from reduction of CO_2_ to establish a hypothetical carbon neutral cycle,
3. decontamination of air and water,
4. low environmental impact for the synthesis of organic compounds,
5. fixation of nitrogen,

These are expected to have a big impact in the new principles of catalysis for circular economy.

**Figure 9:**
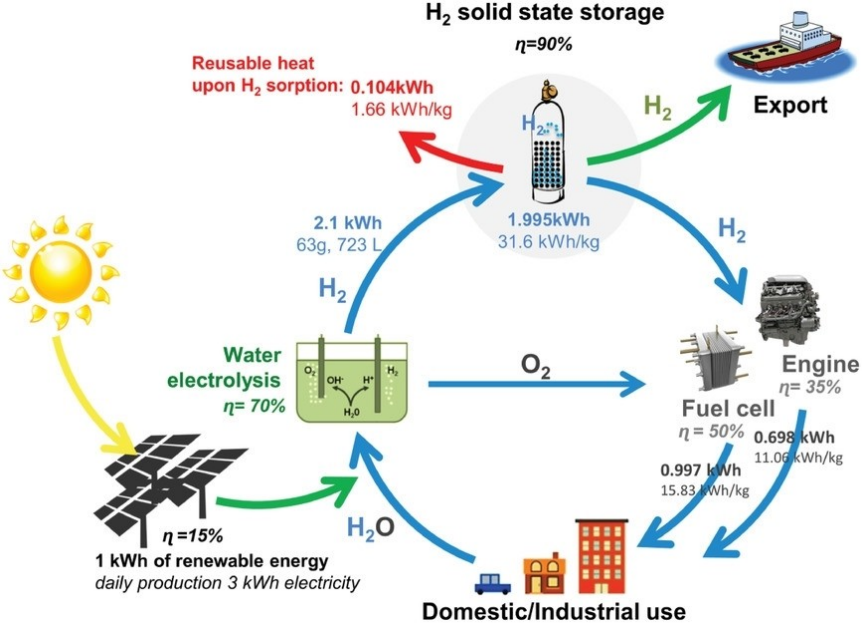
Illustration of the hydrogen renewable energy chain. Reprinted with permissions from Ref. [71] with permission from Wiley, copyright (2019).

Similar conceptual approaches extend to the use of electrocatalysis, and the lion share of studies tackles the same chemical transformations as above, although there are different technical issues. The simpler utilization of the energy input, which does not involve matter‐light interaction, is counterbalanced by the complexity of the electrochemical cell design in relation to the catalytic reaction and all the phenomena associated with electrified elements (i. e. electrodes).^[120]^ Moreover, there is a larger number of components whose optimum integration within the final device needs to be addressed in order to have efficient current circulation and utilization.^[121]^ Without descending into all the technical details, which are beyond the scope of the present perspective, one basic consideration concerning electrocatalytic devices concerns the upstream sustainability of the process, which too often is taken for granted when dealing with electrocatalysis. However, for example in the case of electrolyzers, where an electrochemical potential is applied to drive chemical reactions, the continuous potential difference is ensured by a transducer, which convert other forms of (primary) energy in (secondary) electrochemical energy. In order to have a real impact in current challenges such as global warming and industrial pollution, it is essential that the primary energy input used in electrolyzers originates from renewable sources solar, wind, hydro, geothermal power,^[122]^ rather than fossil fuels. This is a general landscape for the world production and use of electricity for all daily life scopes (not only chemical reactions). It is worth mentioning that combination of two types of energy supply has led to new emerging approaches which better comply with current guidelines on sustainability, for instance use of photo‐electrochemical or photo‐thermal driven processes.^[124]^ Development of materials for storing energy also merits special attention, because energy storage offers a short‐term intermediate and versatile solution to the energy problem, overcoming the issues associated to the direct use of some renewable but fluctuating energy sources such as wind and solar. Storage devices such as batteries, fuel cells, capacitors, flywheels as well as hydrogen storage materials allow the on‐demand energy erogation, and the material used is of central relevance.^[126]^ The properties of the material must be tailored with great care in order to fulfil the requirements of the specific storage technology. As an example, Li‐ion batteries typically suffer from slow charging, quick degradation, poor energy density and conductivity, so that continuous research is carried out on electrodic materials able to enhance charge storage capacity and charge mobility.^[127]^ In this context, the possibility of exploiting nanotechnology has allowed great advances. Two dimensional layered materials consisting of metal dichalcogenides, oxides, nitrides and carbides as well as graphene exhibit high charge mobility and high initial capacities and cycling, although they often decay rapidly, so that research has focused on inorganic heterostructures composed of constituents with opportune properties that can interact synergistically.^[129]^ A popular application for energy storage is that of hydrogen storage materials. In fact, H_2_ bears enormous expectations as the main fuel of future economy, as it is considered as the ideal green energy vector (Figure 9).^[130]^ It has an energy density based on mass (∼147 MJ/kg) which is three times higher than that of gasoline, and its combustion in principle generates only water as the by‐product (in practice, this is strictly true for some kinds of applications, such as fuel cells, but not for other such as direct combustion). These features justify the great efforts in sustainable production of H_2_ through photocatalysis or electrocatalysis, as previously illustrated.

Storage of hydrogen, however, is problematic because its volumetric energy density is low, and current methodologies for storing hydrogen requires compression to pressures as high as 70 MPa, which prevent straightforward use in some applications. Much research has focused on the development of solid state materials able to uptake and release hydrogen reversibly at moderate temperature and pressure, and with high efficiency. Metal alloys and metal organic frameworks (MOF) currently represent two among the most popular choices.^[131]^ An intriguing opportunity for circular economy arises from the possibility of carrying out two reactions in parallel within the same reactor, so doubling the utility of the process. An emblematic case is the application of photocatalysis to drive organic transformation with concomitant release of useful hydrogen which can be separated and stored. A typical organic substrate used for this scope are alcohols, which can be converted into useful carbonyl compounds such as ketones, aldhehydes and carboxylic acids. The required catalyst must be tailored with task‐specific properties and in general includes two or more phases. Chai et al. reported Ni‐modified CdS nanoparticles where the interface between the Ni and the CdS was necessary for efficient catalysis.^[135]^ The approach is made even more sustainable if the alcohol (or in general the organic substrates) derives from biomasses.^[136]^ More complex organic couplings or synthetic processes have also been studied.^[138]^

### Catalyst Recycling and Reuse

**Figure 10:**
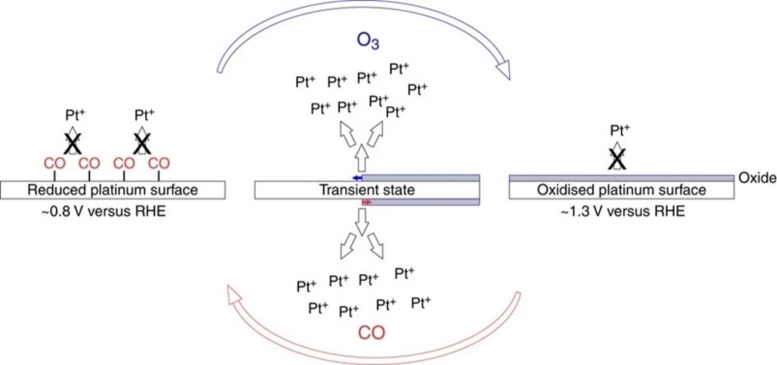
Sketch of the transient process of protective Pt‐oxide growth induced by O_3_ purging (blue arrow) and CO‐induced transient process of oxide removal (red arrow). Reprinted with permissions from Ref. [80] under the terms of the Creative Commons CC BY license.

The final aspect we wish to mention is the recovery of the material itself. Recycling schemes form an important part of the industrial development for cost reduction, but it also plays an important role in the mitigation of materials’ criticality. Recycling is a very general and widespread concept, that should be effected at several levels and for a large variety of products. For instance, there is a massive research on the recycling schemes for plastics, which has notorious consequences either economically than environmentally. However, here we intend to limit only to aspects related to inorganic catalysts recovery, for which the technical strategy is diversified because depends on many factors such as the type of material and the application for which they have been used. Therefore, the cost analysis and economic viability are made on a case‐by‐case basis. Industry makes extensive use of metal catalysts, in many cases consisting of complex composition, which makes the separation and recovery rather challenging. For instance, synthesis of methanol and ammonia involves relies on CuO/ZnO/Al_2_O_3_ catalysts which are recycled applying pyrometallurgical and hydrometallurgical methods. High temperature reduction allows extraction of a good portion of metallic copper (about 66 %), and about 70 % of zinc (separated as ZnO). On the other hand, hydrometallurgical leaching increases the recovered amount of copper up to 98 % of total, while Zn is recycled in the form of ZnAl_2_O_4_. This can be either reused to produce catalysts, or further processed to recover metallic zinc.^[139]^ Even more economic relevance is associated with the recycling of precious metals such as Pt and Pd, which is still based on energy intensive processes due to their corrosion resistance, which often also presents safety issues, typical cases being boiling aqua regia or pressurized alkaline cyanide solutions used in recovery of automotive catalytic converters.^[140]^ Attractive new developments have proposed the use of organic solvents (e. g. mixtures of SOCl_2_ and pyridine) as alternatives to *aqua regia*, where the dissolution occurs through different mechanisms,^[142]^ or ionic liquids for electro‐dissolution and recovery.^[143]^ Still, both approaches do not seem to fully comply with good level of sustainability, being SO_2_ and pyridine hazardous and toxic compounds, and ionic liquids not always economically sustainable. Driven by industry′s strong reliance on such metals, and to guarantee production security in compliance with sustainable guidelines, research is understandably eager to find greener methodologies. This can be achieved only by going deeper into the fundamental knowledge of the materials. Hodnik *et al* investigated the stability of platinum in PEM cells, and acquired important insights, which permitted to conceive a new approach to dissolve Pt under mild conditions of low acid concentration. The key point was the presence of gases like O_3_ or CO, which could interact with Pt and prevent the redeposition of the metal, so facilitating the dissolution through alteration of surface potential (Figure 10).^[144]^

Our group has been interested for several years in the development of new materials for use as three‐way catalysts. Inspired by the use of halides as lixiviants in hydrometallurgy extraction of precious metals, we reported the use of the soft ligand Me_2_dazdt ⋅ 2I_2_ (Me_2_dazdt‐N,N′‐dimethylperhydrodiazepine‐2,3‐dithione) to leach palladium from TWC spent catalysts via formation of a square planar metal complex.^[145]^ Interestingly, such a complex could be not only relevant for recycling of the Pd, but used itself as a precursor to prepare Pd‐based photoreforming catalysts, with a higher H_2_ productivity than commercial Pd.^[146]^ Whenever applicable, this philosophy of “trash to resource”, where the valorization of spent catalyst is performed by direct reuse in catalysis seems to perfectly capture the concept of circular economy.

## Conclusions

Modern times face new and threatening challenges, which demand immediate actions in a holistic sense. Chemistry plays one of the main characters: because most of the climate‐related issues originate from energy intensive chemical processes, only by implementing new sustainable schemes of production will give chemistry the opportunity to redeem itself. New sustainable chemistry will allow the complete abandonment of linear economy and accelerate the awaited circular economy. Heterogeneous catalysis can give tremendous contributions to such a change, and in the present perspective, we present what in our opinion are the modern concepts to be used in the development of catalytic material. But the revolution will have to consider a series of multiple and interconnected aspects, because the chain of events for transition to circular economy overarches many different segments, of scientific, political, ethical and health nature. Herein we highlighted some of the concepts that need to be absorbed during the development of a new inorganic chemistry framework, because they represent (as of today) the most promising strategies to revert on circular economy. Given the complexity of the topic, we selected some of the most general or meaningful examples and provide a critical discussion on them. However, the manuscript has no ambition to be comprehensive, and the aim remains to advertise on recent trends in catalysis with high expectations. The underlined message is that scientists, will need to operate within their scientific competences with a new spirit, and a mind ready to keep updated and adapt to the new discoveries and the fast‐changing needs of our society. New tracks will become available to be explored, and inorganic chemist will have to plunge into it.

## Conflict of Interests

The authors declare no conflict of interest.

1

## Biographical Information

*Michele Melchionna obtained his Ph.D. at the University of Edinburgh, after which he held several positions both in academia (Finland, Czech Republic) and industry (Australia). Since 2013 he has been working at the University of Trieste, where he is currently Associate Professor in Inorganic Chemistry. His research focuses on the design, synthesis, characterization and catalytic activity of new materials for energy and sustainable synthesis applications*.

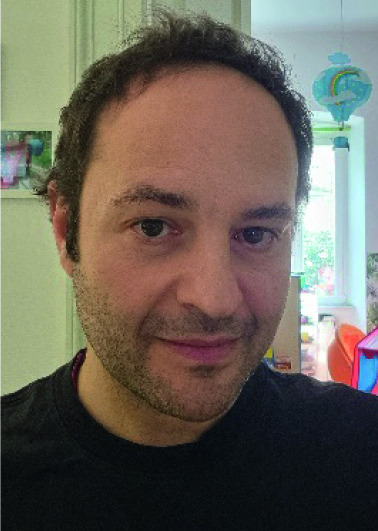

## Biographical Information

*Paolo Fornasiero is Professor of Inorganic Chemistry at the University of Trieste. His scientific interests are in the field of inorganic chemistry, with attention to the design and development of multi‐functional nano‐systems and their advanced applications in energy related processes and environmental heterogeneous catalysis. Since 2022 he is member of the Academia Europaea and from 2021 he is member of the European Academy of Sciences. He received various awards including the 2005 Nasini Medal, the 2013 Chiusoli Medal and the 2022 Malatesta award from the Italian Chemical Society, the 2016 Heinz Heinemann Award from the International Association of Catalysis Societies*.

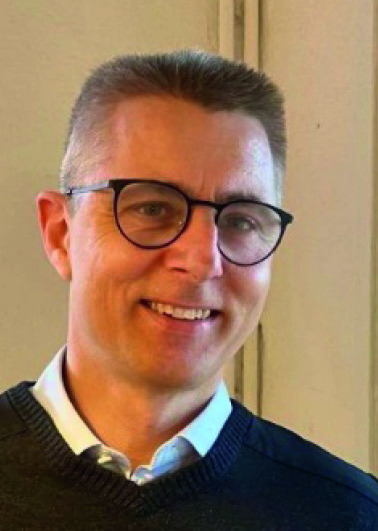

## Data Availability

The data that support the findings of this study are available from the corresponding author upon reasonable request.
